# Editorial Note: Terahertz Irradiation Promotes Angiogenesis in vitro by Enhancing Permeability of the Voltage-Gated Calcium Channel

**DOI:** 10.1371/journal.pone.0352622

**Published:** 2026-06-30

**Authors:** The PLOS One Editors

The *PLOS One* Editors issue this Editorial Note for this article [1] because of concerns related to the peer review process. Readers are encouraged to approach the article [1] with careful consideration.

The Editors do not have concerns regarding the authors’ conduct during the peer review process.
